# ﻿The phylogeny and taxonomy of *Glypholecia* (Acarosporaceae, lichenized Ascomycota), including a new species from northwestern China

**DOI:** 10.3897/mycokeys.98.104314

**Published:** 2023-06-21

**Authors:** An-cheng Yin, Qiu-yi Zhong, Christoph Scheidegger, Ji-zhen Jin, Fiona R. Worthy, Li-song Wang, Xin-yu Wang

**Affiliations:** 1 CAS Key Laboratory for Plant Diversity and Biogeography of East Asia, Kunming Institute of Botany, Chinese Academy of Sciences, Kunming, Yunnan 650201, China; 2 Yunnan Key Laboratory for Fungal Diversity and Green Development, Kunming Institute of Botany, Chinese Academy of Sciences, Kunming 650201, China; 3 Snow and Landscape Research (WSL), Biodiversity and Conservation Biology, Swiss Federal Institute for Forest, Zürcherstrasse 111, 8903 Birmensdorf, Switzerland; 4 Key Laboratory of Plant Stress Research, College of Life Sciences, Shandong Normal University, Jinan, Shandong, 250014, China

**Keywords:** cosmopolitan, lichenized fungi, morphological diversity, phylogenetic analyses, Tibetan Plateau

## Abstract

*Glypholeciaqinghaiensis* An C. Yin, Q. Y. Zhong & Li S. Wang is described as new to science. It is characterized by its squamulose thallus, compound apothecia, ellipsoid ascospores, and the presence of rhizines on the lower surface of the thallus. A phylogenetic tree of *Glypholecia* species was constructed based on nrITS and mtSSU sequences. Two species *G.qinghaiensis* and *G.scabra* are confirmed in China.

## ﻿Introduction

*Glypholecia* Nyl. is a genus of lichenized fungi belonging to Acarosporaceae, Acarosporales, Acarosporomycetidae, Lecanoromycetes, Ascomycota (Nylander 1853; Wijayawardene et al. 2022). The genus *Glypholecia* is characterized by its squamulose-subfoliose, peltate-subumbilicate thallus, compound small apothecia forming multiple structures, and multi-spored asci, generally exceeding 30 spores per ascus. It is therefore distinct from the genus *Acarospora*, which has areolate or squamulose thallus, mostly single or several assembled apothecia, and multi-spored asci, generally exceeding 100 spores per ascus (Ajaj et al. 2007; Sohrabi et al. 2019).

The first known specimen of the genus now designated *Glypholecia*, was collected in the summer of 1810, by Balbis G. B., who was working at the Botanical Gardens of Turin, Italy. Balbis sent this lichen specimen to Germany, where it was received by Funck H. C., who labelled the specimen as *Balbis*. This name was never published. Later, Persoon H. C. examined this same specimen in France, and described it as a new species *Urceolariascabra* Pers. (Persoon 1810).

In 1814, Acharius E. published the lichen species *Lecanorarhagadiosa* Ach (Acharius 1814). In 1850, Schaerer L. E. added an additional lichen species to this genus: *Lecanoragrumulosa* Shaer (Schaerer 1850).

In 1853, Nylander W. established the monotypic genus *Glypholecia*, based on the type species *Glypholeciacandidissima* Nyl. (Nylander 1853). In 1871, Fries T. M. treated these four species names (*Urceolariascabra* Pers., *Lecanorarhagadiosa* Ach, *Lecanoragrumulosa* Shaer and *Glypholeciacandidissima* Nyl.) as synonyms of *Acarosporascabra* (Pers.) Th. Fr. (Fries 1871). In 1892, Müller Arg. treated *Urceolariascabra* and *Acarosporascabra* as synonyms, and designated them as *G.scabra* (Pers.) Müll. Arg. (Müller 1892). The subspecies G.scabravar.candidissima (Nyl.) H. Magn. was published; conserving the name *G.candidissima* Nyl. (Hue 1909; Steiner 1921; Zahlbruckner 1940; Lamb 1963). Recently published phylogenies split Acarosporaceae into 6 main clades, including *Acarospora* (Westberg et al. 2015). Knudsen split the previous *Acarosporaglaucocarpa* group into the North American *Sarcogynecanadensis-wheeleri* clade and the European *Acarosporaglaucocarpa* group (Knudsen et al. 2020). They placed *G.scabra* within the *Sarcogynecanadensis-wheeleri* clade.

Accordingly, most lichenologists accept only one species *G.scabra* (Pers.) Müll. Arg. as belonging to the genus *Glypholecia*. The distribution of *G.scabra* is largely disjunct, with intercontinental populations. It occurs on siliceous rocks in desert, alpine regions, including Europe, Africa, Asia, and North America (Magnusson 1940; Thomson 1979, 1984; Ryan 2002). It exhibits extensive morphological diversity across its range.

Previously, a second species, *G.tibetanica* H. Magn., was described as endemic to China. It has only been recorded from Xizang province, China (Zahlbruckner 1933). However, Obermayer (2004) suggested that *G.tibetanica* might belong to Acarosporanodulosavar.reagens (Zahlbr.) Clauzade and Cl. Roux, which has also been reported from China, based on the K reaction of the cortex. But Magnusson H. appears to have incorrectly reported a ‘C’ reaction (to calcium hypochlorite) as a KOH reaction (potassium hydroxide). According to Zahlbruckner’s (1933) description, the cortex of *G.tibetanica* should show a reddish C+ reaction due to gyrophoric acid, whereas A.nodulosavar.reagens shows a red K+ reaction caused by norstictic acid (Magnusson 1940; Zahlbruckner 1933; Cao and Wei 2009). Unfortunately, the holotype of *G.tibetanica* has been lost, so there are no materials available for further research regarding this putative species.

During the Second Tibetan Plateau Scientific Expedition and Research Program (STEP), we collected numerous lichen specimens from across northwestern China. The expedition included many field surveys across the type locality of *G.tibetanica*, during which we discovered some specimens that conformed to Zahlbruckner’s (1932) description. Our new specimens differ from A.nodulosavar.reagens in the characteristics of their upper cortex, hymenium chemical reaction and their number of ascospores. The molecular sequences obtained from these new specimens demonstrate that they belong to the genus *Glypholecia*, rather than to *Acarospora* (Knudsen et al. 2020). Of these, some samples had squamulose thalli, compound apothecia and white rhizines. These were confirmed as belonging to a new species of the genus *Glypholecia*. In this paper, we describe a new species, *Glypholeciaqinghaiensis*, from northwestern China.

## ﻿Materials and methods

### ﻿Morphological and chemical study

We examined materials of *Glypholecia* from the lichen herbaria of the Kunming Institute of Botany (KUN-L), the College of Life Science and Technology, Xinjiang University (XJU), and the Swiss Federal Institute for Forest, Snow and Landscape Research, Switzerland (WSL). We made morphological observations of the specimens using a Nikon SMZ 745T (Nikon Corp., Tokyo, Japan) dissecting microscope. We cut vertical sections of apothecia and thalli using a razor blade, mounted sections in GAW (glycerol: ethanol: water = 1: 1: 1), then examined them under a Nikon Eclipse 50i stereomicroscope. We measured the average spore size and described sections under both the microscope and stereomicroscope. We photographed all specimens with a Nikon digital camera head DS-Fi2. We identified secondary metabolites by their color reaction coupled with thin-layer chromatography (TLC), using solvent system C (toluene: acetic acid = 85:15), following the methods of Culberson (1970) and Orange et al. (2001).

### ﻿DNA extraction, purification and sequencing

We extracted total genomic DNA from 20 specimens (19 from China and one from Switzerland) using the DNAsecure Plant Kit (Tiangen Biotech, Beijing) following the manufacturer’s protocol. We amplified the internal transcribed spacer regions (nrITS) with the primer pairs ITS1F (Gardes and Bruns 1993) and ITS4 (White et al. 1990). We amplified the mitochondrial small subunit (mtSSU) with primer pairs SSU1 and SSU3R (Zoller et al. 1999). We performed PCR ampliﬁcation with 25 μL volume containing: 12.5 μL 2× MasterMix (0.1 units/μL TaqDNA polymerase, 4 mM MgCl_2_, and 0.4 nM dNTPs; Aidlab Biotechnologies Co. Ltd), 1 μL of each primer, 9.5 μL of ddH_2_O, and 1 μL of DNA, following the PCR settings and primer profile of Zhao et al. (2015). Polymerase chain reaction (PCR) products were sequenced by TsingKe Biological Technology company (Kunming, China).

### ﻿Phylogenetic analysis

We aligned DNA sequences using the program MAFFT v. 7.107 in GENEIOUS v. 8.0.2, setting the following parameters: algorithm = auto; scoring matrix = 200 PAM / k=2; gap open penalty = 1.53; offset value = 0.123 (Katoh et al. 2005). We conducted single-gene analyses to test for potential incongruence among the two-gene fragments, using maximum likelihood (ML) analyses and Bayesian inference (BI). We generated a matrix of *Glypholecia* and its related genera using GENEIOUS v. 8.0.2. In addition to the DNA sequences obtained from our own material, we also downloaded all available sequences of *Glypholecia* from GenBank at the National Center for Biotechnology Information (NCBI, https://www.ncbi.nlm.nih.gov/), and added these to the matrix. We performed ML analyses within RaxML v. 8.2.12 (Stamatakis 2014), using the General Time Reversible model of nucleotide substitution with the gamma model of rate heterogeneity (GTRGAMMA). We selected best partitioning scheme and evolutionary models for two pre-defined partitions using PartitionFinder2 (Lanfear et al. 2016), with greedy algorithm and AICc criterion. We used PhyloSuite (Zhang et al. 2020) inferred Bayesian Inference phylogenies using MrBayes 3.2.6 (Ronquist et al. 2012) under a partition model (2 parallel runs, 10 million generations), for which the initial 25% of sampled data were discarded as burn-in. We inferred support values from the 70% majority-rule tree of all saved trees obtained from 1000 non-parametric bootstrap replicates. We obtained Posterior Probabilities (PPs) from the 95% majority rule consensus tree of all saved trees. We visualized tree files using FigTree 1.4.4.

## ﻿Results

In the present study we generated twenty new nrITS and eighteen new mtSSU sequences. We constructed ML and BI topologies based on these nrITS and mtSSU sequences, and nine additional sequences downloaded from GenBank (Table 1). We used *Pleopsidium* as the outgroup (Crewe et al. 2006; Reeb et al. 2007; Schmull et al. 2011). In the phylogenetic tree, the *Glypholecia* specimens formed a monophyletic lineage, which was divided into two clades, representing *G.scabra* in Clade 2 and the new species *G.qinghaiensis* in Clade 1. The result showed high support for new specimens in this study being assigned to *Glypholecia* (99% ML and 1.00 PP, Fig. 3).

**Table 1.** Specimens and sequences used for phylogenetic analyses. Newly generated sequences are in bold.

Taxon	Locality	Voucher specimens	GenBank number (nrITS)	GenBank number (mtSSU)	References
*G.qinghaiensis*	Ningxia, China	KUN-L 10-0241	**MZ330798**	**OP749902**	–
	Gansu, China	KUN-L 18-58434	**MZ330797**	**OP749903**	–
	Gansu, China	KUN-L 18-59534	**MZ330793**	**OP749907**	–
	Qinghai, China	KUN-L 20-68255	**MZ330789**	**OP749910**	–
	Xinjiang, China	KUN-L 22-71630	**OP749916**	**OP749899**	–
*G.scabra*	Neimenggu, China	XJU 20157514-a	**MZ330786**	–	–
	Gansu, China	KUN-L 18-58747	**MZ330796**	**OP749904**	–
	Qinghai, China	KUN-L 18-59190	**MZ330792**	**OP749906**	–
	Gansu, China	KUN-L 18-58881	**MZ330795**	**OP749905**	–
	Xizang, China	KUN-L 19-65418	**MZ330791**	**OP749908**	–
	Xizang, China	KUN-L 19-66159	**MZ330790**	**OP749909**	–
	Xinjiang, China	KUN-L XY22-856	**OP749911**	**OP749895**	–
	Xinjiang, China	KUN-L XY22-856-2	**OP749912**	**OP749896**	–
	Xizang, China	KUN-L XY22-584	**OP749913**	**OP749894**	–
	Xinjiang, China	KUN-L 22-72868	**OP749914**	**OP749897**	–
	Xinjiang, China	KUN-L 22-71693	**OP749915**	**OP749898**	–
	Xizang, China	KUN-L 22-71500	**OP749917**	**OP749900**	
	Xizang, China	KUN-L 22-71500-2	**OP749918**	**OP749901**	–
	Xizang, China	KUN-L 22-71435	**OP749919**	**OP749893**	–
	Canton of Valais, Switzerland	Scheideg-10522	**MZ330788**	–	–
	Unknown	AFTOL 1008	HQ650722	–	Schmull et al. 2011
	Oppland, Norway	S. Westberg 08-232	LN810811	LN810936	Westberg et al. 2015
*Acarospora* sp.	Gansu, China	Huang Manrong GS157	FJ919810	–	Cao and Wei 2009
*A.placodiiformis*	Spain, Madrid	Westberg 10-211	LN810795	LN810920	Westberg et al. 2015
*A.schleicheri*	Bouches-du-Rhône, France	DUKE Reeb VR5-VII-98/30	DQ525529	–	Reeb et al. 2007
	Sichuan, China	UPS L-070426	LN810800	LN810925	Westberg et al. 2015
	Arizona, USA	UPS L-162697	LN810801	LN810926	Westberg et al. 2015
*Pleopsidiumflavu*m	Steiermark, Austria	UPS L-105590	AY853385	AY853336	Crewe et al. 2006
*P.chlorophanum*	Jämtland, Sweden	UPS L-179248	LN810813	LN810938	Westberg et al. 2015

Our phylogenetic results indicated that *Glypholecia* is monophyletic in China. The genus *Glypholecia* is characterized by its squamulose to crustose thallus, compound apothecia forming multiple structures, multi-spored asci (usually exceeding 30 spores per ascus), spherical small ascospores and C+ red reaction of the cortex due to the presence of gyrophoric acid.

Species of *Glypholecia* were separated into two main clades, as inferred from the phylogenetic tree with strong support. Based on the combination of morphological characters and phylogenetic analysis, we propose a new species in *Glypholecia*. We divide the specimens of the genus *Glypholecia* collected from China into two clades, which correspond to differences in the lower surface morphology of the thallus: the specimens in Clade 1 have ellipsoid ascospores (2.5–3 × 4–6.5 μm), and rhizines on the lower surface of the thallus, whereas Clade 2 contains specimens with spherical ascospores (3–4 µm), and a central holdfast, called an umbilicus. Within Clade 2, our samples of *G.scabra* collected from China were clustered with the European samples, but with some genetic divergence. All *Glypholecia* specimens which we collected from the type locality of “*G.tibetanica*” were monophyletic with *G.scabra*, with a high support value. Within Clade 1, those specimens assigned as *G.qinghaiensis* lack genetic variability within the gene regions included in this study. This might be due to either the sparse population or the shared geography and environment. All of our *G.qinghaiensis* specimens were collected from arid to semi-arid areas of northwestern China, usually growing on sandy rock or sandy soil.

Our phylogenetic analyses showed that a specimen from Gansu province in China, which was identified as *G.scabra* by Cao and Wei (2009), was clustered with *Acarosporaschleicheri* (the type species of *Acarospora*) in Clade 3. Therefore, this specimen’s previous identification as *G.scabra* was incorrect.

### ﻿The new species

#### ﻿ Glypholecia qinghaiensis

Taxon classificationFungiAcarosporalesAcarosporaceae

An C. Yin, Q. Y. Zhong & Li S. Wang, sp. nov.

556592F6-76BF-5278-AAED-14F04F53D61D

839606

Fig. 1 青海聚盘衣

##### Remark.

Resembles *G.scabra*, has abundant and compound apothecia, but differs in having ellipsoid ascospores, rhizines, and a different molecular fingerprint (based on nrITS and mtSSU data).

##### Type.

China, Qinghai Prov., Haixi Mongolian and Tibetan Autonomous Prefecture, Dulan Co., 3066 m, on sandy soil, 2020-09-15, Wang Lisong et al. 20-68255 (holotype – KUN).

***Thallus*** squamulose to squamulose-subfoliose, peltate-subumbilicate, up to 6 mm in diam., 6.5–7 mm thick, with margins sometimes rolling under; surface: upper surface white, pale brown to brown, cracked and wrinkled, usually warty, partly pruinose or occasionally densely pruinose at margins; lower surface white, gray to pale brown, rough, wrinkled, rhizines, umbilicate, 3–6 mm long, attached with a broad; upper cortex: paraplectenchymatous, pale brown, containing calcium oxalate crystals, 30–70 μm thick; medulla: pale, containing calcium oxalate crystals, 125–250 μm thick; hyphae loose, arachnoid, 2.5–3 μm. ***Apothecia*** very common, lecanorine, punctiform when young, but later becoming compound reddish brown to dark brown, becoming lower than the thallus surface when mature; disc with cracks and sometimes tuberculous; paraphyses septate, 2–2.5 μm in diam.; margins concolorous with the thallus; asci: clavate, c. (30–)50-spored; ascospores: ellipsoid, 4–6.5 × 2.5–3 μm, hyaline, thick wall. ***Pycnidia***: rare, flask-shaped. Conidia bacilliform, c. 2–3 × 1 µm.

##### Chemistry.

Hymenium: I+ blue; cortex and medulla: K–, C+ red, KC+ red, P–; secondary metabolites: gyrophoric acid.

##### Ecology and distribution.

Usually on sandy rocks or rarely on soil over rocks; so far only known from Gansu, Ningxia, Qinghai and Xizang provinces in China; growing in arid to semi-arid areas; distributed from 1600 to 4700 m altitude.

##### Etymology.

The epithet “*qinghaiensis*” refers to the holotype locality of the species.

##### Notes.

*Glypholeciaqinghaiensis* can be distinguished from *G.scabra* by having ellipsoid ascospores and rhizines. Phylogenetic analysis in this study supports the separate classification of these two species. This species has only been reported from northwestern China, including Gansu, Ningxia, Qinghai and Xizang provinces.

##### Specimens examined.

China (stored in KUN). Gansu Prov.: Jiuquan City, Yumen City, Yuerhong Vil., 3044 m, on rock, 2018-05-27, Wang Lisong et al. 18-59534; Subei Mongolian Autonomous Co., Suyan Line, 2376m, on soil over rock, 2018-05-23, Wang Lisong et al. 18-58434; Ningxia Prov.: Zhongwei Co., Suwumuyang Site, 1611 m, on rock, 2010-09-19, Niu Dong-Ling et al. 10-0241. Qinghai Prov.: Dulan Co., 3066 m, on sandy rock, 2020-09-15, Wang Lisong et al. 20-68255; Xinjiang Prov.: Wuqia Co., Fossil hill, 2559 m, on sandy rock, 2022-06-26, Wang Lisong et al. 22-71630.

**Figure 1.**
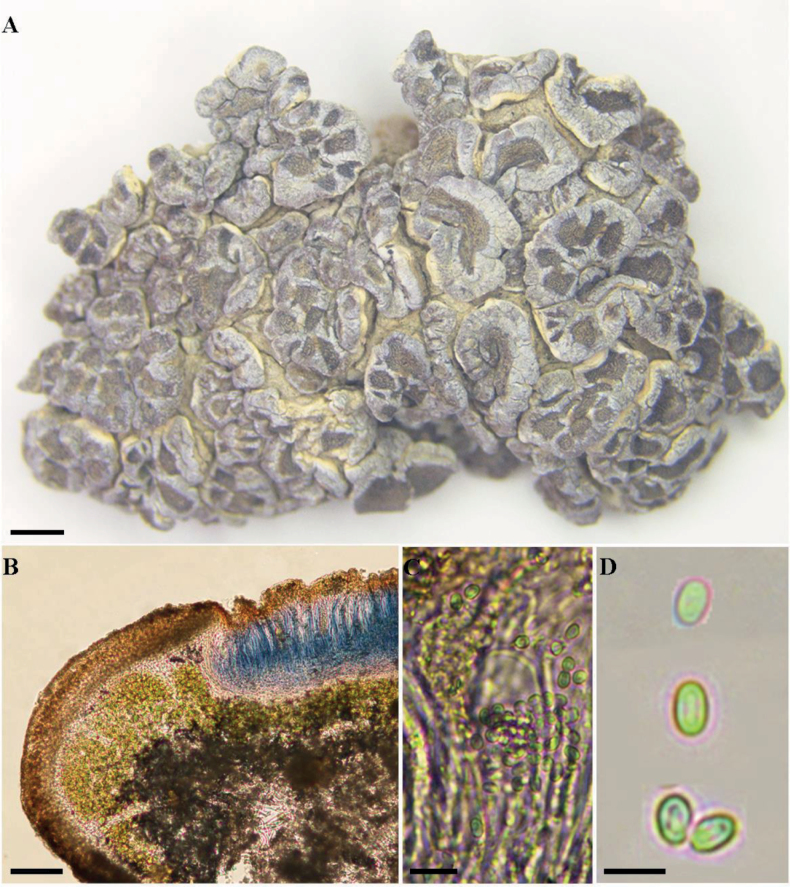
Morphology and anatomy of *Glypholeciaqinghaiensis***A** upper surface of the thallus **B** section of the thallus and apothecia (Lugol’s) **C** paraphyses and asci **D** ascospores. Scale bars: 1 mm (**A**); 50 μm (**B**); 10 μm (**C**); 5 μm (**D**).

### ﻿Species of *Glypholecia* reported in China

#### ﻿ Glypholecia scabra

Taxon classificationFungiAcarosporalesAcarosporaceae

(Pers.) Müll. Arg., Hedwigia 31: 156, 1892.

3103D9F7-EC69-5422-90C5-18AAB1A456DD

≡ Urceolariascabra Pers., Ann. Wetter. Gesellsch. Ges. Naturk. 2: 10, 1810.

##### Type.

Monte Cenisio [in Alps between France and Italy], s. d., Balbis s. n. (not seen).

**Figure 2.**
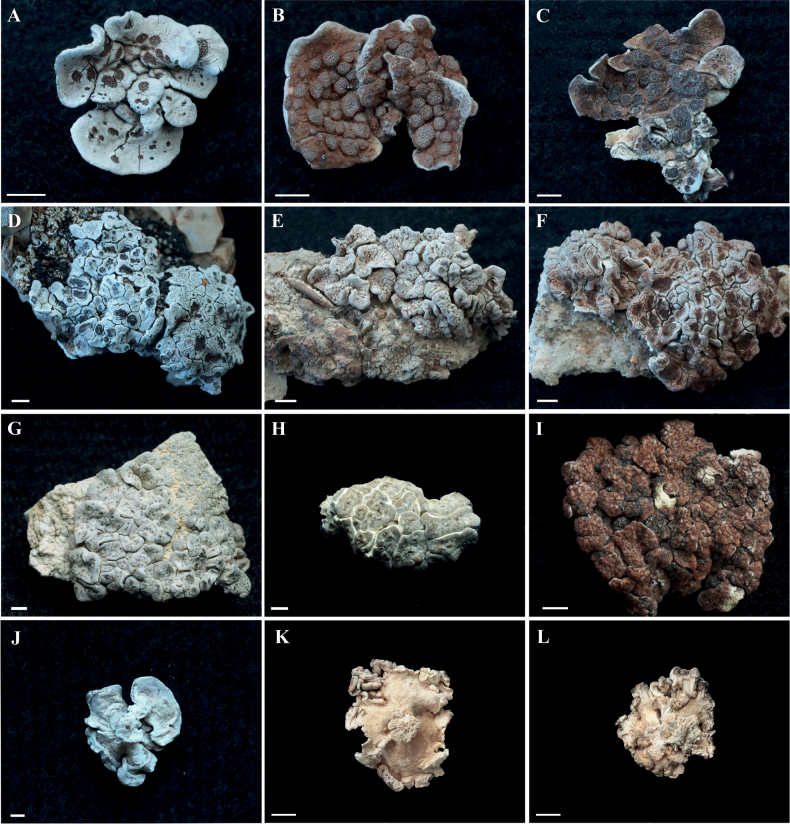
Morphological diversity within the genus *Glypholecia***A–F***Glypholeciascabra*. Differing morphology putatively caused by different habitat conditions, e.g., high or low temperature, aridity, different altitudes (1300–5100 m) **G–I***G.tibetanica*. Collected from type locality, thallus with numerous pycnidia. J-F Umbilicus at the lower surface, formed by fasciculate white rhizines **A, J** Wang Lisong KUN 18-58925 **B** Wang Lisong KUN 18-58814-b **C** Wang Lisong KUN 18-58820 **D** Wang Lisong KUN 19-65418 **E, K** Wang Lisong KUN 18-59346 **F** Wang Lisong KUN 18-58747 **G** Wang Xinyu KUN XY22-854 **H** Wang Lisong KUN 22-71500 **I, L** Wang Xinyu KUN XY22-856. Scale bars: 1 mm.

*Glypholeciascabra* is characterized by its squamulose thallus, abundant and compound apothecia, and umbilicate lower surface, as shown in Fig. 2A–F, J–L. In the Qinghai-Tibetan Plateau, these specimens have an umbilicus at the center of the lower surface, formed by fasciculate white rhizines, which are single or branched, dense or loose. It has a global distribution (see citations above). Within China it has been reported from Gansu (Magnusson 1940; Cao and Wei 2009), Xinjiang (Wang 1985; Abbas et al. 1993; [Bibr B1][Bibr B2]), Ningxia (Liu and Wei 2013) and Xizang (Wei and Jiang 1986) provinces. Delimiting species boundaries can be complicated by the potential role of the environment in shaping morphology. Our phylogenetic study showed that a specimen previously collected and reported from Gansu (GenBank number: FJ919810) did not belong to the genus *Glypholecia*, but should rather be placed within *Acarospora*. We also report a new record for *G.scabra* in Neimenggu province, northern China. For further synonyms and detailed descriptions of *G.scabra*, see Thomson (1979) and Ryan (2002).

**Figure 3.**
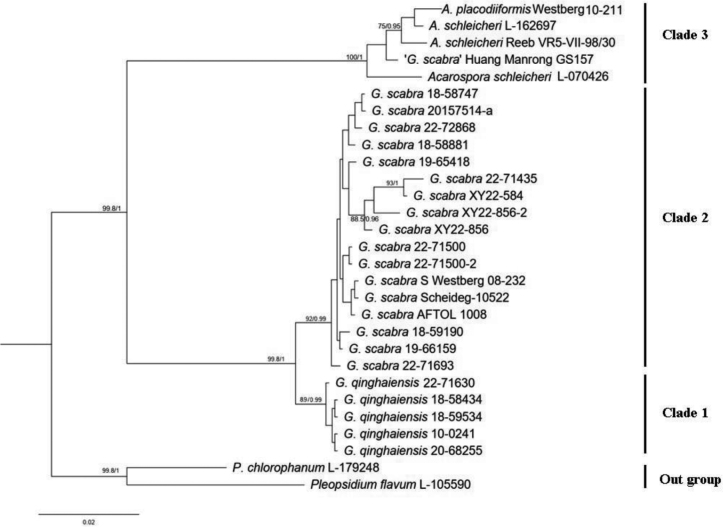
Maximum likelihood (ML) phylogeny of the genus *Glypholecia* and related species of Acarosporaceae, based on nrITS and mtSSU sequences. ML bootstrap value ≥ 70% and posterior probabilities ≥ 0.95 from the Bayesian analysis are displayed adjacent to nodes.

##### Specimens examined.

China. Gansu Prov. (stored in KUN): Zhangye City, Sunan Yugur Autonomous Co., on the way from Sunan to Qilian, hinterland of Qilian Mt., 3958 m, on rock, 2018-05-30, Wang Lisong et al. 18-58881; near Binggoudanxia Geopark, 1984 m, on rock, 2018-05-29, Wang Lisong et al. 18-58747. Qinghai Prov. (stored in KUN): Hainan Tibetan Autonomous Region, Gonghe Co., Heimahe Vil., 3429 m, on rock, 2018-05-19, Wang Lisong et al. 18-59190; Xining City, Huangyuan Co., on the way from Xining to Qinghai Lake, 2476 m, on rock, 2018-05-18, Wang Lisong et al. 18-59094. Neimenggu Prov. (stored in XJU): Alashan League, 1342 m, 2015-08-16, Hurnisa Xahidin 20157514-a. Xinjiang Prov. (stored in XJU): eastern Tianshan Mt., Miquan Tree Farm, 1959 m, 2015-06-28, Hurnisa Xahidin 20155538. Xizang Prov. (stored in KUN): Dingjie Co., Riwu Town, 4848 m, on rock, 2019-07-28, Wang Lisong et al. 19-66159; Cuoqin Co., 5015 m, on rock, 2019-07-20, Wang Lisong et al. 19-65418.

Switzerland (stored by C. Scheidegger). Canton of Valais: Evolène, Mount Le Tsaté., 2492 m, on calcareous rocks, s. d., C. Scheidegger Scheideg-10522.

#### ﻿ Glypholecia tibetanica

Taxon classificationFungiAcarosporalesAcarosporaceae

H. Magn., Feddes Repert. Spec. Nov. Regni veg. 31: 24, 1932.

514C3496-C736-581F-908E-C8E20FE58924

##### Type.

Aksai-Chin-Plateau, [in Xizang Prov., China], (in Botanischer Garten Zürich, holotype, lost).

These specimens were collected by Walter Bosshard in 1927 in Ritu County, Xizang Province, and then reported by H. Magnusson as a new species *G.tibetanica*, characterized by its squamulose thallus, C+ red cortex, abundant black pycnidia and cylindroid conidia, 3.5–4.5 × 1 μm, but the cited type specimen was sterile (Zahlbruckner 1933). Its characteristic of abundant pycnidia is not seen in *G.qinghaiensis*. Obermayer (2004) suggested that *G.tibetanica* might belong to Acarosporanodulosavar.reagens. Alternatively, because the cortex of *G.tibetanica* differs in having a C+ red reaction (versus *A.nodulosa* has C–, K+ red), it might belong to a different species. Therefore, we thoroughly sampled specimens of the genus *Glypholecia* at the type locality of *G.tibetanica.* These new specimens have umbilicate, upper cortex paraplectenchymatous, C+ red, KC+ red, K–, P– in the medulla and contain gyrophoric acid. In contrast, *A.nodulosa* has few rhizines, upper cortex scleroplectenchymatous, C–, K+ yellow turning red, P+ orange-yellow in the medulla, and contains norstictic acid.

We found some *Glypholecia* specimens with numerous pycnidia, as shown in Fig. 2G–H, but rarely with apothecia. Their lower surface usually had fasciculate rhizines aggregated into an umbilicate. Although morphological characters, including the shape (bacilliform), size (3.5–4.5 × 1 µm) of the conidia, and spot reaction (cortex and medulla C+ red) are consistent with those of Zahlbruckner’s *G.tibetanica*, the molecular data show that these specimens instead belong to *G.scabra*. In 2019, we also searched the herbarium of the Zurich Botanical Garden for the holotype of *G.tibetanica* which had been deposited by Walter Bosshard (Zahlbruckner 1933). The holotype could not be located. Therefore, we propose that *G.tibetanica* could potentially be treated as a synonym of *G.scabra*. Further research is required to determine their synonymy. At present, as the holotype could not be examined, the species name *G.tibetanica* should be maintained.

## Supplementary Material

XML Treatment for Glypholecia qinghaiensis

XML Treatment for Glypholecia scabra

XML Treatment for Glypholecia tibetanica

## ﻿Additional information

### Author ORCIDs

An-cheng Yin https://orcid.org/0009-0000-7837-7797

Qiu-yi Zhong https://orcid.org/0000-0001-6256-6083

Christoph Scheidegger https://orcid.org/0000-0003-3713-5331

Ji-zhen Jin https://orcid.org/0009-0005-4351-8162

Fiona R. Worthy https://orcid.org/0000-0003-0042-3110

Li-song Wang https://orcid.org/0000-0003-3721-5956

Xin-yu Wang https://orcid.org/0000-0003-2166-6111

### Data availability

All of the data that support the findings of this study are available in the main text or Supplementary Information.
